# Poly[[aqua­bis­[μ_2_-6-(pyridine-3-car­box­amido)­naphthalene-2-carboxyl­ato]copper(II)] dihydrate]

**DOI:** 10.1107/S1600536812044157

**Published:** 2012-10-31

**Authors:** Yun-Sung Song, Soon W. Lee

**Affiliations:** a Department of Chemistry (BK21), Sungkyunkwan University, Natural Science Campus, Suwon 440-746, Republic of Korea

## Abstract

The title compound, {[Cu(C_17_H_11_N_2_O_3_)_2_(H_2_O)]·2H_2_O}_*n*_, is a two-dimensional polymer. The Cu^2+^ ion lies on the crystallographic twofold axis. The coordination sphere of the Cu^2+^ ion can be described as a distorted square pyramid. All of the H atoms in the amide group and lattice water mol­ecules participate in O—H⋯O or N—H⋯O hydrogen bonding to strengthen the two-dimensioal framework of the polymer.

## Related literature

For coordination polymers based on linking ligands with O- and N-donor atoms, see: Robin & Fromm (2006 ▶). For *d*–*f* coordination polymers based on linking ligands with pyrid­yl–carboxyl­ate terminal ligands, see: Hu *et al.* (2012 ▶); Chen *et al.* (2010 ▶); Tang *et al.* (2010 ▶); Yue *et al.* (2011 ▶); Zhu *et al.* (2010 ▶). For related potential linking ligands, see: Han & Lee (2012 ▶); Zheng & Lee (2012 ▶). For the ligand used for the preparation of the title compound, see: Song & Lee (2012 ▶).
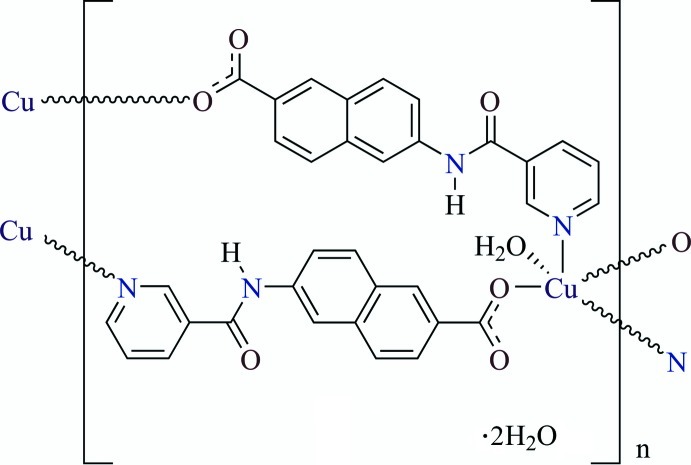


## Experimental

### 

#### Crystal data

[Cu(C_17_H_11_N_2_O_3_)_2_(H_2_O)]·2H_2_O
*M* *_r_* = 700.14
Monoclinic, 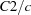

*a* = 29.6255 (8) Å
*b* = 6.8582 (2) Å
*c* = 14.8264 (4) Å
β = 94.728 (3)°
*V* = 3002.14 (14) Å^3^
*Z* = 4
Mo *K*α radiation
μ = 0.79 mm^−1^
*T* = 296 K
0.18 × 0.16 × 0.16 mm

#### Data collection

Bruker APEXII CCD diffractometer
Absorption correction: multi-scan (*SADABS*; Sheldrick, 1996 ▶) *T*_min_ = 0.870, *T*_max_ = 0.884
24367 measured reflections
3705 independent reflections
2462 reflections with *I* > 2σ(*I*)
*R*_int_ = 0.071

#### Refinement

*R*[*F* ^2^ > 2σ(*F* ^2^)] = 0.045
*wR*(*F* ^2^) = 0.095
*S* = 1.00
3705 reflections
234 parameters
H atoms treated by a mixture of independent and constrained refinement
Δρ_max_ = 0.31 e Å^−3^
Δρ_min_ = −0.28 e Å^−3^

### 

Data collection: *APEX2* (Bruker, 2008 ▶); cell refinement: *SAINT* (Bruker, 2008 ▶); data reduction: *SAINT*; program(s) used to solve structure: *SHELXS97* (Sheldrick, 2008 ▶); program(s) used to refine structure: *SHELXL97* (Sheldrick, 2008 ▶); molecular graphics: *SHELXTL* (Bruker, 2008 ▶); software used to prepare material for publication: *SHELXTL*.

## Supplementary Material

Crystal structure: contains datablock(s) I, global. DOI: 10.1107/S1600536812044157/aa2070sup1.cif

Structure factors: contains datablock(s) I. DOI: 10.1107/S1600536812044157/aa2070Isup2.hkl

Additional supplementary materials:  crystallographic information; 3D view; checkCIF report

## Figures and Tables

**Table 1:** Hydrogen-bond geometry (Å, °)

*D*—H⋯*A*	*D*—H	H⋯*A*	*D*⋯*A*	*D*—H⋯*A*
N1—H1⋯O5^i^	0.81 (3)	2.07 (3)	2.857 (4)	164 (3)
O5—H51⋯O2^ii^	0.75 (4)	2.13 (4)	2.861 (3)	164 (4)
O5—H52⋯O3	0.77 (4)	2.04 (4)	2.811 (4)	177 (5)
O4—H4⋯O2^iii^	0.90 (3)	1.95 (3)	2.820 (3)	163 (3)

Symmetry codes: (i) 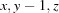; (ii) 
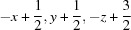
; (iii) 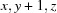.

**Fig. 1.**
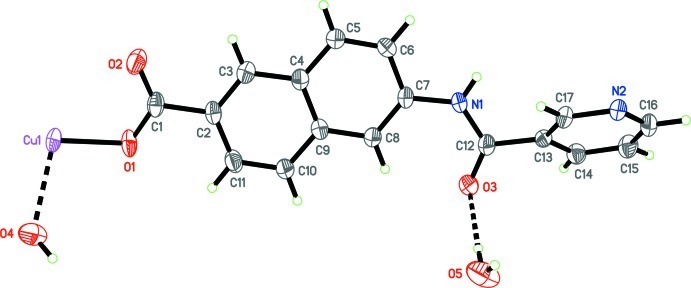
An asymmetric unit of the title compound showing the atomic numbering and 50% probability displacement ellipsoids.

**Fig. 2.**
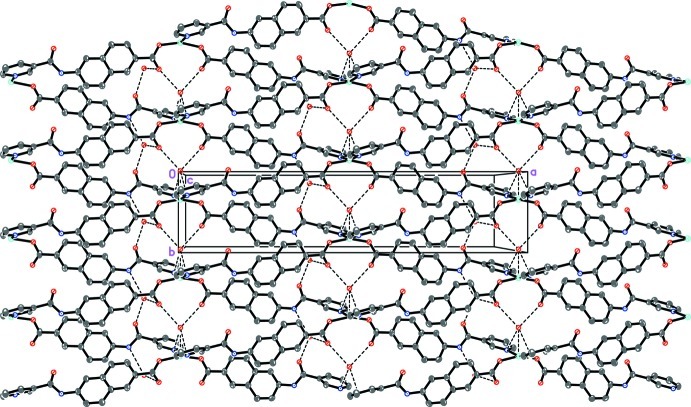
Packing diagram of the title compound, showing a 2-D layer in the [110] direction. Dotted lines represent hydrogen bonds.

## supplementary crystallographic information

### Comment

Coordination polymers are prepared by employing a wide variety of linking ligands possessing pyridyl–pyridyl, pyridyl–amine, furan–furan, thiophene–thiophene, or pyridyl–carboxylate terminals. For instance, bis(pyridyl)- and dicarboxylate-type linking ligands have long been utilized in preparing such polymers (Robin & Fromm, 2006). In particular, those containing the pyridyl–carboxylate terminals are intriguing due to the presence of both a harder carboxylate oxygen donor and a softer pyridyl nitrogen donor in them. The ligands of this type were employed to prepare unique polymers containing both *d*- and *f*-block metals within their frameworks (Hu *et al.*, 2012; Chen *et al.*, 2010; Tang *et al.*, 2010; Yue *et al.*, 2011; Zhu *et al.*, 2010). 6-(Nicotinamido)-2-naphthoic acid (HL) belongs to the pyridyl–carboxylate-type linking ligands, and we recently reported its preparation and structure (Song & Lee, 2012). Our research group also reported the molecular structures of two other related linking ligands (Han & Lee, 2012; Zheng & Lee, 2012). We report herein the structure of a two-dimensional Cu polymer of the HL ligand, which is the first *d*-block coordination polymer of this ligand.

Fig. 1 shows an asymmetric unit of the title polymer, which consists of one half Cu^2+^ ion, one 6-(nicotinamido)-2-naphthoato ligand (*L*), one half aqua ligand, and one lattice water molecule. The Cu1 and O4 atoms lie on the crystallographic twofold axis, and the remaining atoms occupy general positions. The Cu^2+^ ion is coordinated to two oxygen atoms from two ligands and two nitrogen atoms from another two ligands to form a distorted square plane. The Cu1···O4 length (2.490 (3) Å), which is represented by a dotted line in Fig. 1, is extremely long, considering the covalent radii of Cu (1.28 Å) and O (0.66 Å) atoms. The van der Waals radii of Cu and O atoms are 1.40 and 1.52 Å, respectively, and therefore the Cu1···O4 bond may be best described as a strong van der Waals contact. Consequently, the coordination sphere of the Cu^2+^ ion may be thought of as square pyramidal, if the Cu1···O4 van der Waals contact is included. The molecular plane, defined by the two O and two N atoms, is extremely distorted from the planarity with the average atomic displacement of 0.328 (1) Å. Two carboxylate oxygen atoms act differently; one (O1) is coordinated to the Cu^2+^ ion and the other (O2) acts as a H-bond acceptor. The terminal carboxylate and pyridyl groups are bonded to the Cu^2+^ ions, indicating that this ligand behaves as a linking ligand. The amide group (–CONH–) does not coordinate to the metal ion, and the carbonyl oxygen (O3) acts as a H-bond acceptor and the N–H bond behaves as a H-bond donor. In fact, all of the hydrogen atoms in the amide group and lattice water molecule participate in the hydrogen bonds of the O–H···O or N–H···O types (Table 1). Fig. 2 shows a projection of the title polymer along the *c*-axis. The repeat unit consists of four ligands and four Cu^2+^ ions. This unit contains 56 atoms (4 Cu^2+^ ions and 52 ligand atoms) with the Cu^2+^···Cu^2+^ separation of 15.2045 (4) Å. The repeat units are connected by the ligands to form a 2-D layer in the [110] direction.

### Experimental

A mixture of Cu(NO_3_)_2_^.^3H_2_O (48.3 mg, 0.2 mmol) and 6-(nicotinamido)-2-naphthoic acid (58.4 mg, 0.2 mmol) in H_2_O (20 ml) was sealed in a 24 ml Teflon-lined vessel. The reaction mixture was heated 150 °C for 72 h and then slowly air-cooled to room temperature for 24 h. The resulting green crystals were isolated by filtration, washed by methanol (10 ml × 3), and then air-dried to give the title compound (19 mg, 0.027 mmol, 27% yield). mp: 586–589 K. IR (KBr, cm^-1^): 3474 (w), 2897 (w), 2633 (w), 2383 (w), 2298 (w), 2084 (w), 1805 (w), 1660 (*m*), 1580 (*m*), 1483 (*m*), 1353 (*m*), 1204 (w), 1108 (w), 1049 (w), 951 (w), 886 (w), 824 (w), 772 (w), 749 (w), 702 (w), 625 (w), 457 (w).

### Refinement

C-bound H atoms were positioned geometrically [C—H = 0.93 Å] and allowed to ride on their parent atoms, with *U*_iso_(H) = 1.2*U*_eq_(C). H-atoms participating in the H-bonds were located in a difference Fourier map and refined freely.

### Crystal data

**Table:**

[Cu(C_17_H_11_N_2_O_3_)_2_(H_2_O)]·2H_2_O	*F*(000) = 1444
*M**_r_* = 700.14	*D*_x_ = 1.549 Mg m^−^^3^
Monoclinic, *C*2/*c*	Mo *K*α radiation, λ = 0.71073 Å
Hall symbol: -C 2yc	Cell parameters from 3663 reflections
*a* = 29.6255 (8) Å	θ = 2.8–26.3°
*b* = 6.8582 (2) Å	µ = 0.79 mm^−^^1^
*c* = 14.8264 (4) Å	*T* = 296 K
β = 94.728 (3)°	Block, green
*V* = 3002.14 (14) Å^3^	0.18 × 0.16 × 0.16 mm
*Z* = 4	

### Data collection

**Table:**

Bruker APEXII CCD diffractometer	3705 independent reflections
Radiation source: sealed tube	2462 reflections with *I* > 2σ(*I*)
Graphite monochromator	*R*_int_ = 0.071
φ and ω scans	θ_max_ = 28.3°, θ_min_ = 1.4°
Absorption correction: multi-scan (*SADABS*; Sheldrick, 1996)	*h* = −38→39
*T*_min_ = 0.870, *T*_max_ = 0.884	*k* = −9→8
24367 measured reflections	*l* = −19→19

### Refinement

**Table:**

Refinement on *F*^2^	Primary atom site location: structure-invariant direct methods
Least-squares matrix: full	Secondary atom site location: difference Fourier map
*R*[*F*^2^ > 2σ(*F*^2^)] = 0.045	Hydrogen site location: inferred from neighbouring sites
*wR*(*F*^2^) = 0.095	H atoms treated by a mixture of independent and constrained refinement
*S* = 1.00	*w* = 1/[σ^2^(*F*_o_^2^) + (0.0323*P*)^2^ + 3.3539*P*] where *P* = (*F*_o_^2^ + 2*F*_c_^2^)/3
3705 reflections	(Δ/σ)_max_ < 0.001
234 parameters	Δρ_max_ = 0.31 e Å^−^^3^
0 restraints	Δρ_min_ = −0.28 e Å^−^^3^

### Special details

**Table:**

Geometry. All s.u.'s (except the s.u. in the dihedral angle between two l.s. planes) are estimated using the full covariance matrix. The cell s.u.'s are taken into account individually in the estimation of s.u.'s in distances, angles and torsion angles; correlations between s.u.'s in cell parameters are only used when they are defined by crystal symmetry. An approximate (isotropic) treatment of cell s.u.'s is used for estimating s.u.'s involving l.s. planes.
Refinement. Refinement of *F*^2^ against ALL reflections. The weighted *R*-factor *wR* and goodness of fit *S* are based on *F*^2^, conventional *R*-factors *R* are based on *F*, with *F* set to zero for negative *F*^2^. The threshold expression of *F*^2^ > σ(*F*^2^) is used only for calculating *R*-factors(gt) *etc*. and is not relevant to the choice of reflections for refinement. *R*-factors based on *F*^2^ are statistically about twice as large as those based on *F*, and *R*- factors based on ALL data will be even larger.

### Fractional atomic coordinates and isotropic or equivalent isotropic displacement parameters (Å^2^)

**Table:**

	*x*	*y*	*z*	*U*_iso_*/*U*_eq_	
Cu1	0.5000	0.15958 (7)	0.7500	0.02730 (14)	
O1	0.43940 (5)	0.1123 (3)	0.69567 (11)	0.0339 (5)	
O2	0.43602 (6)	−0.1766 (3)	0.76371 (12)	0.0404 (5)	
O3	0.14263 (6)	0.0207 (3)	0.50325 (12)	0.0388 (5)	
O4	0.5000	0.5226 (5)	0.7500	0.0458 (8)	
O5	0.11363 (10)	0.3690 (4)	0.58013 (19)	0.0586 (7)	
N1	0.15983 (7)	−0.2657 (4)	0.57653 (15)	0.0298 (5)	
N2	0.02186 (6)	−0.2919 (3)	0.62447 (13)	0.0248 (5)	
C1	0.41869 (8)	−0.0433 (4)	0.71535 (16)	0.0286 (6)	
C2	0.36998 (8)	−0.0565 (4)	0.67842 (16)	0.0278 (6)	
C3	0.34545 (8)	−0.2230 (4)	0.69110 (17)	0.0315 (6)	
H3	0.3597	−0.3288	0.7207	0.038*	
C4	0.29901 (8)	−0.2372 (4)	0.66017 (16)	0.0277 (6)	
C5	0.27238 (8)	−0.4042 (4)	0.67428 (18)	0.0348 (7)	
H5	0.2857	−0.5120	0.7037	0.042*	
C6	0.22799 (8)	−0.4089 (4)	0.64557 (17)	0.0337 (6)	
H6	0.2111	−0.5201	0.6555	0.040*	
C7	0.20668 (8)	−0.2475 (4)	0.60056 (16)	0.0274 (6)	
C8	0.23130 (8)	−0.0832 (4)	0.58502 (16)	0.0286 (6)	
H8	0.2174	0.0225	0.5550	0.034*	
C9	0.27774 (8)	−0.0746 (4)	0.61459 (16)	0.0269 (6)	
C10	0.30397 (8)	0.0933 (4)	0.60178 (18)	0.0337 (7)	
H10	0.2906	0.2001	0.5716	0.040*	
C11	0.34869 (8)	0.1023 (4)	0.63273 (17)	0.0336 (7)	
H11	0.3652	0.2148	0.6234	0.040*	
C12	0.13114 (8)	−0.1337 (4)	0.53628 (16)	0.0268 (6)	
C13	0.08218 (8)	−0.1870 (4)	0.53737 (16)	0.0247 (5)	
C14	0.05252 (8)	−0.1765 (4)	0.45994 (16)	0.0295 (6)	
H14	0.0624	−0.1341	0.4053	0.035*	
C15	0.00809 (9)	−0.2305 (4)	0.46597 (17)	0.0329 (7)	
H15	−0.0123	−0.2295	0.4147	0.040*	
C16	−0.00604 (8)	−0.2859 (4)	0.54859 (16)	0.0292 (6)	
H16	−0.0362	−0.3207	0.5518	0.035*	
C17	0.06539 (8)	−0.2429 (4)	0.61731 (16)	0.0262 (6)	
H17	0.0852	−0.2471	0.6692	0.031*	
H1	0.1478 (10)	−0.368 (5)	0.588 (2)	0.055 (11)*	
H4	0.4757 (10)	0.599 (6)	0.754 (3)	0.083 (13)*	
H51	0.1036 (13)	0.338 (6)	0.623 (2)	0.072 (14)*	
H52	0.1223 (14)	0.273 (7)	0.561 (3)	0.088 (17)*	

### Atomic displacement parameters (Å^2^)

**Table:**

	*U*^11^	*U*^22^	*U*^33^	*U*^12^	*U*^13^	*U*^23^
Cu1	0.0144 (2)	0.0424 (3)	0.0249 (2)	0.000	0.00091 (16)	0.000
O1	0.0186 (8)	0.0527 (14)	0.0301 (9)	−0.0109 (9)	0.0001 (7)	0.0015 (9)
O2	0.0268 (9)	0.0467 (14)	0.0464 (11)	0.0048 (10)	−0.0051 (8)	0.0007 (10)
O3	0.0274 (10)	0.0329 (12)	0.0565 (12)	−0.0030 (9)	0.0062 (9)	0.0119 (10)
O4	0.052 (2)	0.032 (2)	0.0547 (19)	0.000	0.0111 (16)	0.000
O5	0.0766 (18)	0.0398 (17)	0.0638 (17)	−0.0107 (13)	0.0319 (14)	−0.0065 (13)
N1	0.0185 (11)	0.0281 (14)	0.0426 (13)	−0.0052 (11)	0.0013 (9)	0.0033 (11)
N2	0.0186 (10)	0.0288 (14)	0.0269 (10)	0.0003 (9)	0.0015 (8)	−0.0006 (9)
C1	0.0190 (12)	0.0431 (19)	0.0237 (12)	−0.0003 (13)	0.0028 (10)	−0.0078 (12)
C2	0.0188 (12)	0.0366 (17)	0.0281 (13)	−0.0046 (12)	0.0028 (10)	−0.0042 (12)
C3	0.0233 (13)	0.0352 (18)	0.0356 (14)	0.0019 (12)	0.0003 (11)	0.0004 (12)
C4	0.0219 (12)	0.0310 (16)	0.0299 (13)	−0.0020 (12)	0.0001 (10)	−0.0020 (11)
C5	0.0260 (14)	0.0308 (17)	0.0467 (16)	−0.0020 (12)	−0.0024 (12)	0.0081 (13)
C6	0.0278 (14)	0.0278 (16)	0.0450 (15)	−0.0059 (12)	−0.0003 (12)	0.0078 (13)
C7	0.0177 (12)	0.0326 (16)	0.0317 (13)	−0.0017 (12)	0.0021 (10)	−0.0005 (12)
C8	0.0220 (12)	0.0290 (16)	0.0343 (13)	−0.0011 (12)	−0.0003 (10)	0.0041 (12)
C9	0.0228 (12)	0.0299 (16)	0.0278 (12)	−0.0038 (11)	0.0012 (10)	−0.0008 (11)
C10	0.0256 (13)	0.0307 (17)	0.0437 (15)	−0.0045 (12)	−0.0031 (11)	0.0074 (12)
C11	0.0256 (13)	0.0376 (19)	0.0372 (14)	−0.0094 (12)	0.0001 (11)	0.0040 (12)
C12	0.0205 (12)	0.0313 (17)	0.0289 (12)	−0.0009 (12)	0.0040 (10)	−0.0020 (12)
C13	0.0204 (12)	0.0205 (15)	0.0332 (13)	0.0012 (11)	0.0031 (10)	−0.0024 (11)
C14	0.0276 (13)	0.0322 (17)	0.0286 (12)	−0.0012 (13)	0.0024 (10)	0.0016 (12)
C15	0.0281 (14)	0.0411 (18)	0.0281 (13)	−0.0006 (12)	−0.0061 (11)	0.0007 (12)
C16	0.0188 (12)	0.0349 (18)	0.0336 (13)	−0.0014 (11)	0.0003 (10)	−0.0013 (11)
C17	0.0187 (12)	0.0330 (16)	0.0265 (12)	0.0013 (11)	−0.0007 (10)	−0.0003 (11)

### Geometric parameters (Å, º)

**Table:**

Cu1—O1	1.9337 (16)	C4—C9	1.424 (4)
Cu1—O1^i^	1.9337 (16)	C5—C6	1.349 (3)
Cu1—N2^ii^	2.0476 (19)	C5—H5	0.9300
Cu1—N2^iii^	2.0476 (19)	C6—C7	1.414 (4)
O1—C1	1.277 (3)	C6—H6	0.9300
O2—C1	1.246 (3)	C7—C8	1.372 (4)
O3—C12	1.227 (3)	C8—C9	1.410 (3)
O4—H4	0.90 (3)	C8—H8	0.9300
O5—H51	0.75 (4)	C9—C10	1.410 (4)
O5—H52	0.77 (4)	C10—C11	1.367 (3)
N1—C12	1.347 (3)	C10—H10	0.9300
N1—C7	1.410 (3)	C11—H11	0.9300
N1—H1	0.81 (3)	C12—C13	1.497 (3)
N2—C16	1.341 (3)	C13—C17	1.377 (3)
N2—C17	1.345 (3)	C13—C14	1.389 (3)
N2—Cu1^iv^	2.0476 (19)	C14—C15	1.377 (3)
C1—C2	1.503 (3)	C14—H14	0.9300
C2—C3	1.374 (4)	C15—C16	1.380 (3)
C2—C11	1.405 (4)	C15—H15	0.9300
C3—C4	1.417 (3)	C16—H16	0.9300
C3—H3	0.9300	C17—H17	0.9300
C4—C5	1.416 (4)		
			
O1—Cu1—O1^i^	160.69 (12)	C8—C7—C6	120.0 (2)
O1—Cu1—N2^ii^	93.07 (7)	N1—C7—C6	116.2 (2)
O1^i^—Cu1—N2^ii^	90.06 (7)	C7—C8—C9	120.1 (2)
O1—Cu1—N2^iii^	90.06 (7)	C7—C8—H8	120.0
O1^i^—Cu1—N2^iii^	93.07 (7)	C9—C8—H8	120.0
N2^ii^—Cu1—N2^iii^	161.31 (12)	C8—C9—C10	121.8 (2)
C1—O1—Cu1	119.48 (17)	C8—C9—C4	119.8 (2)
H51—O5—H52	104 (4)	C10—C9—C4	118.4 (2)
C12—N1—C7	128.7 (2)	C11—C10—C9	121.4 (3)
C12—N1—H1	114 (2)	C11—C10—H10	119.3
C7—N1—H1	118 (2)	C9—C10—H10	119.3
C16—N2—C17	117.1 (2)	C10—C11—C2	120.7 (3)
C16—N2—Cu1^iv^	123.25 (15)	C10—C11—H11	119.6
C17—N2—Cu1^iv^	119.30 (15)	C2—C11—H11	119.6
O2—C1—O1	124.2 (2)	O3—C12—N1	124.8 (2)
O2—C1—C2	120.2 (2)	O3—C12—C13	121.1 (2)
O1—C1—C2	115.6 (2)	N1—C12—C13	114.1 (2)
C3—C2—C11	119.3 (2)	C17—C13—C14	118.5 (2)
C3—C2—C1	120.2 (2)	C17—C13—C12	119.8 (2)
C11—C2—C1	120.5 (2)	C14—C13—C12	121.7 (2)
C2—C3—C4	121.5 (3)	C15—C14—C13	118.4 (2)
C2—C3—H3	119.2	C15—C14—H14	120.8
C4—C3—H3	119.2	C13—C14—H14	120.8
C5—C4—C3	123.1 (2)	C14—C15—C16	119.5 (2)
C5—C4—C9	118.3 (2)	C14—C15—H15	120.2
C3—C4—C9	118.7 (2)	C16—C15—H15	120.2
C6—C5—C4	120.9 (3)	N2—C16—C15	122.8 (2)
C6—C5—H5	119.6	N2—C16—H16	118.6
C4—C5—H5	119.6	C15—C16—H16	118.6
C5—C6—C7	121.0 (3)	N2—C17—C13	123.6 (2)
C5—C6—H6	119.5	N2—C17—H17	118.2
C7—C6—H6	119.5	C13—C17—H17	118.2
C8—C7—N1	123.8 (2)		

Symmetry codes: (i) −*x*+1, *y*, −*z*+3/2; (ii) −*x*+1/2, *y*+1/2, −*z*+3/2; (iii) *x*+1/2, *y*+1/2, *z*; (iv) *x*−1/2, *y*−1/2, *z*.

### Hydrogen-bond geometry (Å, º)

**Table:**

*D*—H···*A*	*D*—H	H···*A*	*D*···*A*	*D*—H···*A*
N1—H1···O5^v^	0.81 (3)	2.07 (3)	2.857 (4)	164 (3)
O5—H51···O2^ii^	0.75 (4)	2.13 (4)	2.861 (3)	164 (4)
O5—H52···O3	0.77 (4)	2.04 (4)	2.811 (4)	177 (5)
O4—H4···O2^vi^	0.90 (3)	1.95 (3)	2.820 (3)	163 (3)

Symmetry codes: (ii) −*x*+1/2, *y*+1/2, −*z*+3/2; (v) *x*, *y*−1, *z*; (vi) *x*, *y*+1, *z*.

## References

[bb1] Bruker (2008). *APEX2*, *SAINT* and *SHELXTL* Bruker AXS Inc., Madison, Wisconsin, USA.

[bb2] Chen, M. S., Su, Z., Chen, M., Chen, S. S., Li, Y. Z. & Sun, W. Y. (2010). *CrystEngComm*, **14**, 3267–3276.

[bb3] Han, S. H. & Lee, S. W. (2012). *Acta Cryst.* E**68**, o294. 10.1107/S1600536811056212 PMC3274987 22346932

[bb4] Hu, S., Sheng, T., Wen, Y., Fu, R. & Wu, X. (2012). *Inorg. Chem. Commun.* **16**, 28–32.

[bb5] Robin, A. Y. & Fromm, K. M. (2006). *Coord* *Chem* *Rev* **250**, 2127–2157

[bb6] Sheldrick, G. M. (1996). *SADABS* University of Göttingen, Germany.

[bb7] Sheldrick, G. M. (2008). *Acta Cryst.* A**64**, 112–122. 10.1107/S0108767307043930 18156677

[bb8] Song, Y.-S. & Lee, S. W. (2012). *Acta Cryst.* E**68**, o1978. 10.1107/S1600536812024051 PMC3393255 22807812

[bb9] Tang, Y. Z., Wen, H. R., Cao, Z., Wang, X. W., Huang, S. & Yu, C. L. (2010). *Inorg. Chem. Commun.* **13**, 924–928.

[bb10] Yue, S. T., Wei, Z. Q., Wang, N., Liu, W. J., Zhao, X., Chang, L. M., Liu, Y. L., Mo, H. H. & Cai, Y. P. (2011). *Inorg. Chem. Commun.* **14**, 1396–1399.

[bb11] Zheng, Z. N. & Lee, S. W. (2012). *Acta Cryst.* E**68**, o774. 10.1107/S1600536812006472 PMC3297845 22412648

[bb12] Zhu, L. C., Zhao, Y., Yu, S. J. & Zhao, M. M. (2010). *Inorg. Chem. Commun.* **13**, 1299–1303.

